# Genomic Epidemiology of Clinical *Brucella melitensis* Isolates from Southern Israel

**DOI:** 10.3390/microorganisms10020238

**Published:** 2022-01-22

**Authors:** Bar Zilberman, Yair Motro, Orli Sagi, David Kornspan, Shalom Ben-Shimol, Michael Gdalevich, Yael Yagel, Nadav Davidovitch, Boris Khalfin, Peter Rabinowitz, Lior Nesher, Itamar Grotto, Svetlana Bardenstein, Jacob Moran-Gilad

**Affiliations:** 1 Faculty of Health Sciences, Ben Gurion University of the Negev, Beer Sheva 84105, Israel; Barsch@post.bgu.ac.il (B.Z.); motroy@post.bgu.ac.il (Y.M.); Orlisa@clalit.org.il (O.S.); shalomb2@clalit.org.il (S.B.-S.); michael.gdalevich@bsh.health.gov.il (M.G.); Ygrushka@gmail.com (Y.Y.); nadavd@bgu.ac.il (N.D.); boriskh83@gmail.com (B.K.); nesherke@bgu.ac.il (L.N.); grotto@bgu.ac.il (I.G.); 2 Soroka University Medical Center, Beer Sheva 84101, Israel; 3 Brucellosis Lab, OIE, FAO Reference Laboratory, Kimron Veterinary Institute, Bet Dagan 50250, Israel; Davidko@moag.gov.il (D.K.); Svetab@moag.gov.il (S.B.); 4 Southern District Health Office, Ministry of Health, Beer Sheva 84104, Israel; 5 Department of Environmental and Occupational Health Sciences, School of Public Health, University of Washington, Seattle, WA 98195, USA; peterr7@uw.edu

**Keywords:** zoonosis, *Brucella melitensis*, WGS, cgMLST, cgSNPs

## Abstract

Brucellosis, a zoonosis mainly transmitted by consumption of unpasteurized dairy products, is endemic in Southern Israel, mainly among the Bedouin Arab population. However, the genomic epidemiology of *B. melitensis* in this region has not yet been elucidated. A cohort of brucellosis cases (*n* = 118) diagnosed between 2017–2019 was studied using whole-genome sequencing (WGS). Phylogenetic analyses utilized core genome MLST (cgMLST) for all local isolates and core genome SNPs for 347 human-associated *B. melitensis* genomes, including Israeli and publicly available sequences. Israeli isolates formed two main clusters, presenting a notable diversity, with no clear dominance of a specific strain. On a global scale, the Israeli genomes clustered according to their geographical location, in proximity to genomes originating from the Middle East, and formed the largest cluster in the tree, suggesting relatively high conservation. Our study unveils the genomic epidemiology of *B. melitensis* in Southern Israel, implicating that rather than a common source, the transmission pattern of brucellosis among Bedouin communities is complex, predominantly local, and household-based. Further, genomic surveillance of *B. melitensis* is expected to inform future public health and veterinary interventions and clinical care.

## 1. Introduction

Brucellosis is a zoonotic disease caused by Gram-negative coccobacilli that belong to the genus *Brucella*. These microorganisms can infect domestic animals, including sheep, goats, cows, and camels, causing significant animal morbidity and agricultural damages. Humans are typically infected through the consumption of contaminated dairy products. Brucellosis is usually not transmitted from person to person, although transmission via blood transfusion, bone marrow transplantation, or vertically has been reported [1,2].

Many parts of the world are endemic for brucellosis, including Africa, Latin America, Central Asia, the Middle East including the Mediterranean Basin, although reported incidence and prevalence vary widely from country to country [3]. The burden of disease in low-income countries has led the World Health Organization (WHO) to classify brucellosis as one of the world’s leading “neglected zoonotic diseases” [4]. In developed countries, brucellosis is generally considered an occupational disease since it mainly affects abattoir workers, veterinarians, laboratory technicians, farmers and livestock producers or is associated with international travel or import foods. In Israel, the disease is prevalent among Arab and Druze populations [5] and is solely caused by *B. melitensis*, as *B. abortus* has been eliminated. Most brucellosis cases occur in Southern Israel, where it affects the Bedouin Arab population, a semi-nomadic tribal society, harboring different kinds of livestock within the household, and is associated with traditional consumption of unregulated and unpasteurized, usually homemade, dairy products.

Brucellosis has variable and often nonspecific clinical manifestations. These include systemic symptoms such as fever, chills, malaise, weight loss, and musculoskeletal pain. Brucellosis can also manifest as an organ-specific disease, such as sacroiliitis, arthritis, vertebral osteomyelitis, and epididymo-orchitis [6]. The involvement of the musculoskeletal system is the most common complication of brucellosis, while other life-threatening complications, such as meningitis and endocarditis, are less common [7]. Therefore, the mortality rate of properly treated brucellosis is usually low [8]. Despite varying rates of organ involvement, factors that predict disease manifestations are yet to be identified. 

The classification of *Brucella* to species and biovars is performed by traditional phenotypic characterization of lipopolysaccharide antigen, phage typing, dye sensitivity, CO_2_ requirement, H_2_S production, and metabolic properties [9]. Such a phenotypic testing approach allows cluster analysis and bio-typing within the genus *Brucella* [10]. The results of the classical bio-typing schemes categorize *B. melitensis* into three biovars. This methodology is of limited value and consistency and does not provide sufficient typing resolution for epidemiological purposes [11]. Moreover, these methods suffer from inconsistencies and require bio-safe handling of live bacteria in a BSL-3 laboratory. For this reason, PCR-based typing is increasingly used as an alternative to culture-dependent typing methods [12]. 

In recent years, new typing methods, including genome-based approaches that allow an accurate differentiation between *Brucella* isolates and the creation of subtyping schemes of this pathogen, have been developed [13,14]. Multilocus variable number of tandem repeats analysis (MLVA) has long been the standard typing tool for brucellosis and is still being used in many countries. However, this typing method has several weaknesses related to the nature of variable-number tandem repeats (VNTRs), technical laboratory requirements, and insufficient resolution [12,15,16,17]. Consequently, the analysis of whole-genome sequences (WGS) of *Brucella* is advantageous for investigating the genomic characteristics of the organism in addition to molecular surveillance purposes [18]. In the context of zoonotic diseases, WGS can assist in linking human, animal, and environmental isolates as part of epidemiological investigations with an unprecedented resolution at the local scale [19,20,21,22]. The usefulness of WGS for genotyping and characterizing brucellosis outbreaks was recently shown for a brucellosis outbreak related to camel milk consumption in Israel [23]. Nevertheless, only a limited number of studies have reported whole-genome comparisons and phylogenetic analyses of *Brucella*. We have recently reported a pilot analysis of 27 whole genomes from human patients in Southern Israel, showing the potential of this approach [24], which is further expanded in the current paper.

This study uses a much larger number of *Brucella* genomes to expand previous analyses to explore the genomic epidemiology of brucellosis in Southern Israel in a local and global context. We applied WGS, a tool having greater typing resolution, to comprehensively study the genomic characteristics and examine the genotype–phenotype association for clinical isolates of *B. melitensis* in Southern Israel during 2017–2019.

## 2. Materials and Methods

### 2.1. Research Setting

The Negev region of Southern Israel roughly covers 60% of Israel’s territory. A significant part of the population inhabiting this area is the Bedouin Arab tribes, a society in transition from a semi-nomadic to a modern lifestyle. The Bedouin partly live-in permanent settlements and partly in villages scattered throughout the desert, characterized by temporary housing lacking infrastructure. The latter is commonly associated with tribal settlement. Members of Bedouin communities frequently raise sheep, cattle, and camels and consume raw unpasteurized dairy products. Such widespread traditional and unregulated consumption of dairy products contribute to the endemicity of brucellosis in the Negev region. 

Soroka University Medical Center (SUMC) is the sole hospital serving the Bedouin communities in the Negev region and therefore provides care for most of the brucellosis cases in the region, allowing for comprehensive molecular epidemiology of local disease spread and association of clinical data with pathogen genomics. 

### 2.2. Collection of Clinical and Epidemiological Data

This study protocol was approved by the SUMC Research Ethics committee (#SOR 0292-17). Clinical and epidemiological information was retrospectively retrieved from electronic medical records of the SUMC and then coded and de-identified, leading to a total of 118 culture-positive brucellosis cases admitted between 2017 and 2019. Medical record data were abstracted using a case report form (CRF) that contained variables such as demographics, clinical data, potential exposures, laboratory findings, treatment data, the clinical course of the disease (acute, reinfection or relapse), and outcome. 

### 2.3. Bacterial Dataset

The genomic dataset for this study included genomes of *B. melitensis* isolates recovered from clinical samples at the microbiology laboratory of SUMC between 2017 and 2019. Overall, we included *Brucella* isolates from 118 culture-positive patients (see Supplementary Table S1a), and thus, our study is based on a homogenous cohort of proven cases, excluding cases diagnosed based solely on serology. All study isolates were submitted to the National Reference Laboratory for brucellosis at the Kimron Veterinary Institute, Ministry of Agriculture and Rural Development, as mandated by law and where bio-typing was performed in addition to the analysis mentioned above. 

### 2.4. DNA Extraction, Library Preparation, and WGS of Israeli Isolates 

DNA was extracted from clinical *Brucella* isolates using a two-step approach (heat killing at 80 °C for 10 min, followed by extraction using the DNeasy Blood and Tissue kit (Qiagen, Hilden, Germany) according to the manufacturer’s instructions. DNA concentration and purity were measured using a Qubit fluorometer with a dsDNA high-sensitivity assay kit (Invitrogen, Waltham, MA, USA) and a NanoDrop™ One/One^C^ Microvolume UV-Vis Spectrophotometer (Thermo Fisher Scientific, Waltham, MA, USA). The genomic libraries were constructed using the Nextera FLEX library preparation kit (Illumina Inc., San Diego, CA, USA), according to the manufacturer’s instructions, followed by sequencing on Illumina sequencers producing 150–250 bp paired-end reads. 

### 2.5. Bioinformatics Analysis

Our analysis of the Israeli genomes was complemented by including publicly available human *B. melitensis* sequences that were downloaded from the NCBI, Sequence Read Archive (SRA) database (https://www.ncbi.nlm.nih.gov/sra/, accessed on 1 May 2020) along with the available metadata. We searched all sequences of *B. melitensis* isolates that had been generated by Illumina sequencing. This search allowed the selection of sequences that were generated using comparable technology. We only included public *Brucella* genomes representing isolates from humans for which paired-end raw sequence data were available, for a total of 230 genomes, using the reads download component of the Flowcraft pipeline (release 1.3.1, https://github.com/assemblerflow/flowcraft, accessed on 1 March 2020, using default parameters, unless stated otherwise). 

All sequencing reads passed QC using FastQC (v0.11.7) https://www.bioinformatics.babraham.ac.uk/projects/fastqc/ (accessed on 1 March 2020), with a minimum sequencing depth 20x, and were identified as *B. melitensis*, while also checking for contamination, using Kraken2 [25] with the minikraken2_v1_8GB database (ftp://ftp.ccb.jhu.edu/pub/data/kraken2_dbs/, accessed on 1 March 2020).

### 2.6. Ad Hoc Core Genome MLST Analysis of Israeli Isolates

We studied genetic relatedness among Israeli *B. melitensis* isolates using core genome MLST (cgMLST), which has been successfully used for creating genomic typing schemes for various organisms, including *B. melitensis* [26,27]. All 118 sequences, were subject to QC, trimming and de novo assembly (see Supplementary Table S1b) using the INNUca pipeline https://github.com/B-UMMI/INNUca (accessed on 1 March 2020) with Trimmomatic (v0.36) [28], SPADes (v3.12.0) [29] and Pilon (v1.23) [30]. Genome assemblies have been deposited in ENA under the bioproject PRJEB48426. All assembled genomes were checked for quality using QUAST [31] (V.5.0.2) and were in silico sequence-typed using the tool mlst (v2.17.6) https://github.com/tseemann/mlst (accessed on 1 February 2020) with the *Brucella* spp. 9-loci scheme from pubMLST (1 May 2020) [32,33] (see Supplementary Table S1b). An ad hoc cgMLST was generated using chewBBACA (v2.1.0) [34] (with a Prodigal [35] training file for the reference genome *B. melitensis* 16M, and including loci with 95% genome presence), with the final ad hoc cgMLST scheme consisting of 2926 loci. A minimum spanning tree (MST) was generated (with MSTreeV2) and visualized from the ad hoc cgMLST scheme for all 118 isolates using GrapeTree [36].

### 2.7. Global B. melitensis cgSNPs Phylogeny

Since the Brucella genus is highly conserved, there is a need to analyze the data at the maximal resolution, and therefore, in the analysis of the global isolates, we used the SNPs calling method. Compared to cgMLST, this method can include intergenic regions and may account for a larger number of genetic differences since each allelic difference could represent multiple differing nucleotides within the same gene [37]. 

A core genome SNP-calling analysis was thus performed to evaluate the genetic relatedness among *347 B. melitensis* genomes. Raw WGS reads (Illumina paired-end) for human-associated *B. melitensis* isolates (see Supplementary Table S2a) were downloaded from NCBI using the SRA toolkit (v2.9.6) https://github.com/ncbi/sra-tools (accessed on 1 February 2020) and subjected to QC using the methods mentioned above (see Supplementary Table S2b). All sequences were in silico sequence-typed using SRST2 (v0.2.0) [38] with the pubMLST scheme for Brucella spp. (May 2020), including *B. melitensis* reference strain 16M complete genome (accession: GCF_000007125.1) using Snippy (v4.6.0) https://github.com/tseemann/snippy (accessed on 1 February 2020) (with default parameters and “--mincov 2”). Core genome SNPs were then determined using a snippy core, with masking of phage sites that were obtained for the reference genome from the PHASTER database (https://phaster.ca/, accessed on 1 March 2020). For the core genome SNPs analysis, only 347 (230 public genomes together with 117 Israeli genomes) genomes that aligned more than 80% to the reference genome were included [39]. Finally, recombination sites were also masked using Gubbins (v2.4.1) [40]. An MST was generated (using the MSTreeV2 method) and visualized from the final masked cgSNPs alignment (consisting of 234 core SNPs) with GrapeTree (v1.5.0) [36].

## 3. Results

### 3.1. Clinical and Epidemiological Characterization of Study Cohort

The study included 118 culture-positive brucellosis patients admitted to the hospital during the years 2017–2019. The distribution of cases across study years was 33, 63, and 38 cases, respectively. Among brucellosis patients, 71% were males, and 56% were under 18 years old. Most of the patients were Bedouin (97%), living in more than 20 different settlements across the Bedouin community in the Negev. The primary culture source of isolates was blood culture (98%), while in the remaining cases, isolates were recovered from an abscess or synovial fluid culture.

Of the various clinical manifestations examined, headaches were reported in 31.3% of patients, night sweats in 16%, and weight loss in 14.4%. Fever for more than seven days was reported by 36.4% of cases; musculoskeletal complaints were reported by 36.4% of patients. Clinical findings such as lymphadenopathy, splenomegaly, and frank arthritis were observed in 9.3%, 1.7%, and 8.6%, respectively. One case of epididymo-orchitis was noted, and no cases of endocarditis or neurobrucellosis were noted. 

The percentage of patients who required hospitalization following a diagnosis of brucellosis was 59.5% of whom 79% were adults. The duration of hospitalization ranged from 1 to 20 days with a mean of 4 ± 3.48 (median 3 days), and 15.3% were hospitalized more than four days. In terms of disease course, the vast majority of patients presented with a primary infection (93%), and only a minority presented with apparent reinfection or relapse of a previous disease. 

### 3.2. Local Phylogeny of Brucella melitensis in Southern Israel

**Figure 1:**
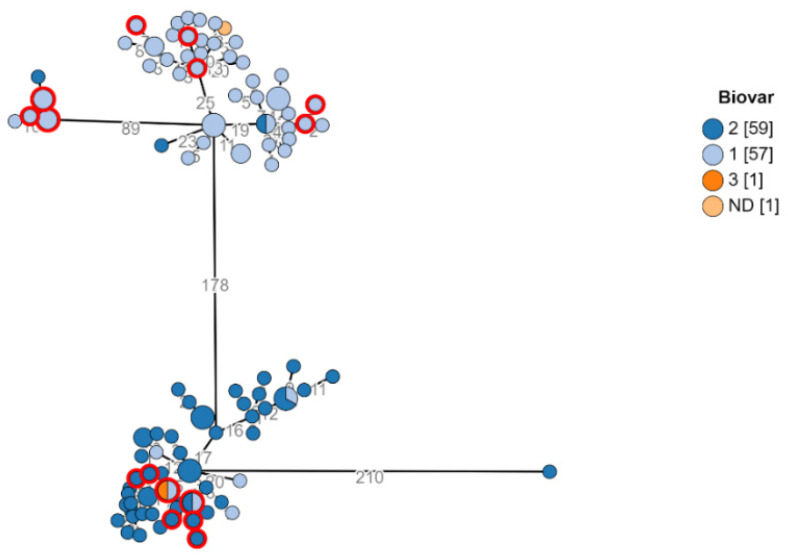
Biovars of *Brucella melitensis* in Southern Israel. Minimum spanning tree showing phylogenetic analysis of *B. melitensis* isolates from Southern Israel. Study isolates (*n* = 118) ad hoc cgMLST for a core genome of 2926 loci. Each node represents a genome, and numbers account for the number of differing alleles between nodes (not to scale). Every node represents an isolate. Each color represents a different Biovar. Nodes representing isolates from the city Rahat are marked in red circles as an example of isolates scattering among the two clusters. Each color represents a different Bio-variant. Multicolored nodes represent different genomes with no variation.

The phylogenetic analysis of the Israeli isolates was conducted using the ad hoc core genome MLST method. Creating a cgMLST scheme for the 118 Israeli *B. melintesis* isolates involved identifying a core set of loci (2926) present in at least 95% of the 118 genomes. The isolates were all sequence-typed as ST8 and belonged to biovar 1, 2, and 3 in 48.3%, 50%, and 0.85% of all cases, respectively. This analysis showed a distinct division into two major clusters in the MST, where the two main biovars were evident within the two main clusters (Figure 1).

**Figure 2:**
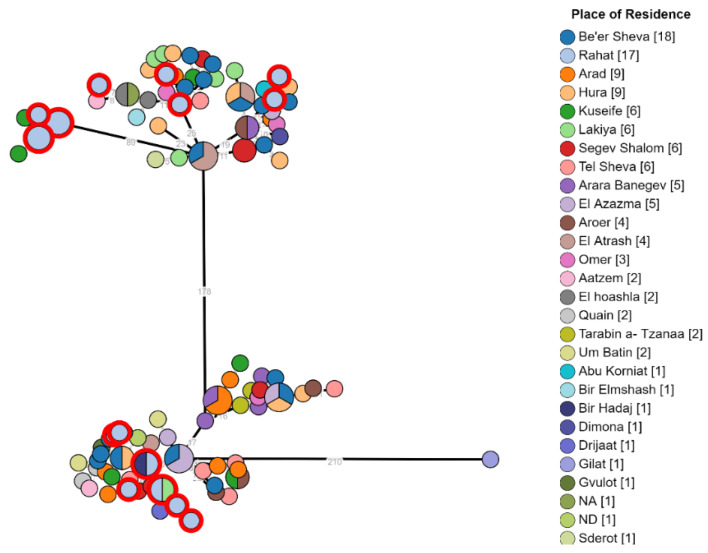
Phylogeny of *Brucella melitensis* isolates from different demographic areas in the Negev. *B. melitensis* was cultured and isolated from 118 patients. The phylogenetic analysis had been conducted using the ad hoc core genome MLST method as mentioned above. In the minimum spanning tree, each node represents a genome, and the numbers between indicate the number of varying alleles between the pairs of genomes. Nodes are colored according to isolate place of residency; multicolored nodes represent different genomes with no variation. Numbers in square brackets indicate the total number of isolates from each location.

The examination of the geographical distribution of isolates from major Bedouin cities such as Rahat, Lakiya, Hura, and Kuseife, or non-permanent villages showed a non-specific distribution across the two branches. In addition, isolates from the same city were observed over both clusters (Figure 2). This finding indicates that there is probably not one common source responsible for most brucellosis morbidity in the Negev and intermixing of strains by location suggests complex epidemiology in the region that involves multiple exposures and transmission events between infected animals and humans across the entire region.

**Figure 3:**
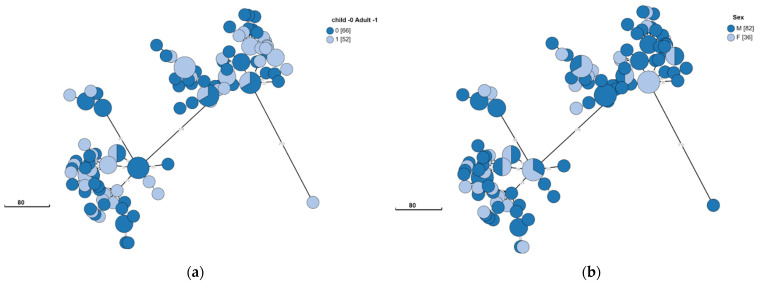

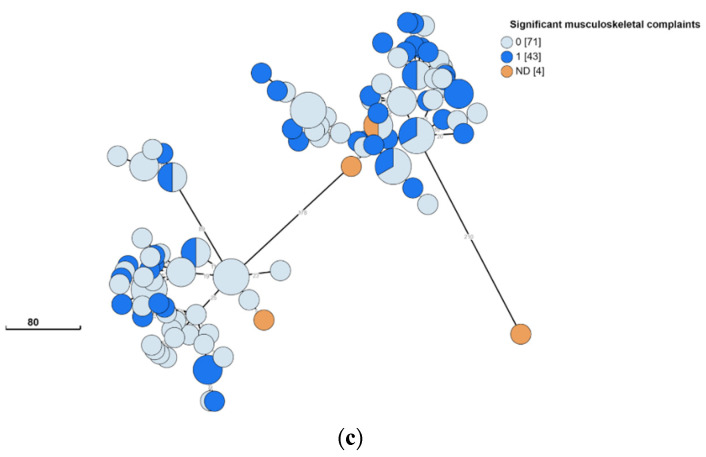
Clinical and epidemiological characteristics of *Brucella melitensis* in Southern Israel. *B. melitensis* was cultured and isolated from 118 patients. The phylogenetic analysis had been conducted using the ad hoc core genome MLST method as mentioned above. The minimum spanning tree shows the phylogenetic analysis of *B. melitensis* isolates from Southern Israel. Each node represents a genome, and numbers account for the number of differing alleles between nodes (not to scale). Each color represents an age (child or adult) (**a**), sex (F/M) (**b**), and significant musculoskeletal complaints (0—no; 1—yes; ND—not detected) (**c**). Numbers in square brackets indicate the total number of isolates from each characteristic.

Isolates originating in children and adults were evenly distributed across the two clusters (Figure 3A) and isolates also did not appear to segregate according to gender (Figure 3B). Thus, no specific disease-causing strains appeared to correlate with age or gender. Musculoskeletal complaints were the most common clinical symptom reported. No specific strain appeared to correlate with the presence or absence of this presentation (Figure 3C). 

### 3.3. Global Phylogeny of B. melitensis Human Isolates

**Figure 4:**
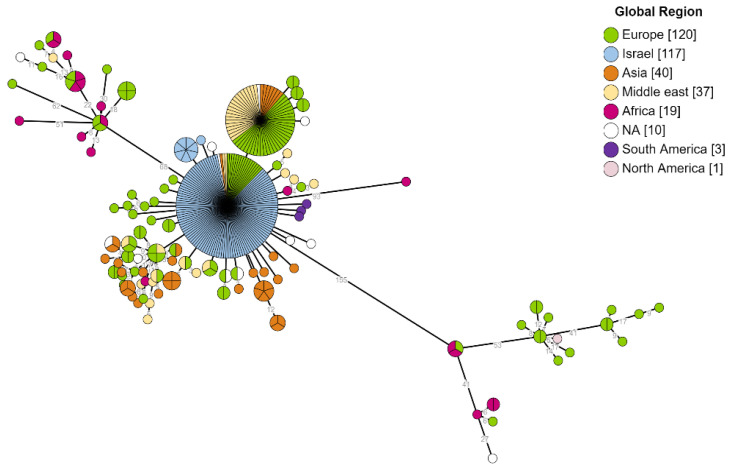
Global phylogeny of *B. melitensis* hosted by human. Phylogenetic analysis utilizing SNP calling of 234 core SNPs from 347 *B. melitensis* genomes. The presented MST tree shows a notable diversity between isolates originating in different global regions. The branch labels correspond to genetic diversity and the node’s color to the global region of the isolate.

Core genome SNP analysis included a total of 347 genomes (117 Israeli and 230 public genomes) isolated from humans. This analysis is based on 234 core SNPs. The resulting MST displayed three major phylogenetic clusters expressing a pronounced genomic diversity. The peripheral clusters included a small number of genomes from all genomes that were analyzed, 13 in the upper cluster and 16 in the lower cluster, mainly from Europe and Africa. Isolates from Europe have been found scattered throughout the MST, possibly representing cases associated with international travel or migration. In contrast, the main cluster contained most of the analyzed genomes that originated from Israel, the Middle East, and Asia. The Israeli genomes were the largest group in the central cluster, without scattering among other clusters. Israeli isolates constituted more than 40% of all human genomes in the public domain due to the contribution of the current study to the global dataset. The clustering of local isolates emphasizes their relative genetic conservation and genomic proximity compared to the overall global view. Moreover, Israeli strains clustered to those originating from the Middle East and Asia (Figure 4).

## 4. Discussion

Brucellosis, considered a neglected zoonosis acquired from contaminated food products, remains a public health concern worldwide. In this study, we describe the phylogeny of *B. melitensis* in Southern Israel. Using WGS, our study reveals a marked diversity of *B. melitensis* isolates in the Negev region, an endemic region, implicating the local transmission pattern among the Bedouin community. Moreover, intermixing of strains by location suggests complex epidemiology in the region that involves the movement of infected animals and persons. However, all isolates from the region clustered together and appeared relatively conserved from a global perspective.

In recent years, there has been a notable increase in the global application of WGS for studying the genomic epidemiology of *B. melitensis*. In fact, recent studies have confirmed that WGS is now universally accepted as the ultimate typing tool for bacterial pathogens. By using a whole-genome SNP-based approach, Tan et al. studied the native geographical origin of the pathogen and provided a basis for the reconstruction of the history of the global spread of *B. melitensis* [41]. Georgi et al. reported a detailed genetic investigation of *B. melitensis* strains isolated from human cases in Germany [42]. Based on these findings, we examined the phylogeographical origin of the pathogen isolated in our area and found that all Israeli isolates were associated with the Middle East cluster, as could be expected considering their origin. Our analysis further broadens the global phylogenetic analysis using the whole-genome SNP-based approach by including 347 *B. melitensis* human-associated isolates, including our local genomes together with the publicly available ones. Unlike the findings presented in previous studies, which were mostly conducted on isolates from Europe and might not reflect the geographic location of primary acquisition, in our study, all clinical cases were native to Southern Israel and not related to tourism or immigration. Our analysis corroborates previous studies, suggesting the WGS-based phylogeny is a practicable and applicable high-resolution tool for typing this organism.

Janowicz and colleagues characterized the topology of the *B. melitensis* phylogeny according to four evident phylogeographical lineages: west Mediterranean, East Mediterranean, the Americas, and African [26]. Our phylogenomic analysis of the local isolates originating from Southern Israel found they belong phylogeographically to the East Mediterranean lineage, which is consistent with Israel’s location in the Middle East and the genetic proximity to genomes originating in Asia. This finding indicates that the Israeli isolates are relatively conserved in the global perspective. Moreover, their study supported previous estimates assuming that the West Mediterranean lineage was the first to diverge from the East Mediterranean lineage [43] and suggested that the global spread of the bacterium began as a result of the intercontinental trade of goats and sheep or their infected products to different regions in the world [26,43]. These statements are consistent with the findings of our study, which indicate the source of the disease in Israeli genomes is only from our region, suggesting the transmission pattern of the disease in our region is predominantly local and household-based in contrast to isolates originating in Europe, which showed greater heterogeneity. This reflects the spread of the pathogen across Europe is primarily associated with tourism or migration of infected individuals or imported food or animal trade. 

Another difference in the phylogenetic characterization of the Israeli genomes found in our work is the association with Biovar. In contrast to publicly available genomes, of which Biovar 3 is the most common [26], our cohort is comprised of two other co-dominant biovars, 1 and 2, and only one case out of our local 118 genomes belonged to Biovar 3. While each of the two biovars was predominantly assigned to a cluster in the phylogenetic tree, some intermixing did occur, suggesting WGS cluster types are not predictive of the biovar. 

Pisarenko et al. performed a global phylogeographic analysis of *B. melitensis*, defined polymorphisms specific for the strains of each of the five genotypes and sub-genotypes found [43], and suggested future research should involve enough verified metadata in order to increase the resolution of the method. Indeed, our study consists of a homogenous three-year cohort of culture-positive brucellosis patients, in which we examined a phylogenetic analysis of human cases together with multiple epidemiological and clinical variables and investigated the possible correlation of certain disease phenotypes with specific infecting strains. In our study, most of the patients were admitted due to a suspected zoonotic manifesting as an acute febrile syndrome, with brucellosis being one of the possible etiologies. The majority of cases have thus been diagnosed as inpatients and discharged after several days of initial intravenous therapy if their clinical conditions allowed while ensuring outpatient treatment continuity. No clear correlation was identified between infecting *B. melitensis* strains and common clinical manifestations, such as musculoskeletal involvement, nor any correlation between infecting strain and host characteristics, such as age and gender. We assume that due to hospitalization, prompt diagnosis and effective parenteral treatment, the number of cases with life-threatening complications (endocarditis/neurobrucellosis) is very low. Due to the observed low proportion of complications as well as adverse outcomes, such as relapsing infection, our study was underpowered and could not identify any phylogenomic correlations associated with severe disease, despite this being a large cohort study of brucellosis patients, as compared to previous studies.

The cgMLST method has been comparable to SNP analysis in Europe and is expected to replace the MLVA-16 typing method of *B. melitensis* [27]. The cgMLST scheme has been proposed as a standard tool for outbreak investigations in human and animal brucellosis and identified countries of the Middle East as the most probable source of origin of the majority of the strains [27,44]. Our cgMLST analysis demonstrated a marked diversity of *B. melitensis* isolates within the Negev region, a finding which suggests that multiple strains are harbored within the reservoir animals across the region and that cases of human infection occur across large geographic and community settings through multiple local exposures rather than a common source. While no common source is responsible for the significant morbidity in the Negev region, small clusters of genetically related isolates, involving 2–3 patients, that also cluster in place and time have been identified, suggesting localized outbreaks did indeed occur, which were possibly related to domestic and household exposure or local trade. In the presence of multiple strains affecting different localities and tribes, a comprehensive and cross-cutting public intervention is warranted. 

This study has several limitations. It is retrospective and therefore clinical and epidemiological information is limited to what was documented in patient records. Since we included only culture-proven brucellosis cases, it is likely that many brucellosis cases were not included in the study as they were only serology-confirmed, and thus, our sample may not be representative. Finally, our study could not assess severe clinical manifestations due to their low frequency. 

## 5. Conclusions

In conclusion, WGS analysis demonstrated the local genetic diversity of the organism and its relative conservation in a global phylogeny context. In addition, we studied a multi-year cohort of culture-proven cases that not only provided a novel representation of the epidemiology in the region but also allowed assessing the correlation between genotype and clinical phenotype in an unprecedented manner. However, no clear correlations were identified. Future studies should involve an in-depth analysis of virulence factors in order to identify genetic bacterial traits associated with severe or complicated brucellosis. In addition, other factors such as human genetic susceptibility to infection or the immune response to brucellosis and their impact on the disease course should be investigated. Our findings carry implications for public health and animal health interventions in the region, suggesting that the implementation of WGS could assist control efforts of brucellosis.

## Data Availability

Whole genomes produced by this research are being deposited into NCBI Genbank. Reads are available in BioProject PRJEB48426.

## Supplementary Materials

The following supporting information can be downloaded at: https://www.mdpi.com/article/10.3390/microorganisms10020238/s1, Table S1: Israeli *Brucella melitensis* isolates included in cgMLST analysis. Table S2: Global *Brucella melitensis* isolates included in cgSNPs analysis hosted by human.
